# Targeting nanoparticles to lung cancer-derived A549 cells based on changes on interstitial stiffness in biomimetic models

**DOI:** 10.1016/j.isci.2024.111015

**Published:** 2024-09-23

**Authors:** Afia Ibnat Kohon, Kun Man, Ala Hessami, Katelyn Mathis, Jade Webb, Joanna Fang, Parsa Radfar, Yong Yang, Brian Meckes

**Affiliations:** 1 Department of Biomedical Engineering, University of North Texas, 3940 N Elm St., Denton, TX 76207, USA; 2 BioDiscovery Institute, University of North Texas, 1155 Union Circle, Denton, TX 76203-5017, USA

**Keywords:** Health sciences, Biological sciences, Applied sciences

## Abstract

The mechanical properties and forces of the extracellular environment modulate alveolar epithelial cell behavior. To model cancer/fibrosis associated stiffening and dynamic stretch, a biomimetic device was developed that imitates the active forces in the alveolus, while allowing control over the interstitial matrix stiffness. Alveolar epithelial A549 cancer cells were cultured on the devices and their transcriptome was profiled with RNA sequencing. Pathway analysis showed soft materials upregulated the expression of proteoglycans associated with cancer. Consequently, liposomes were modified with peptides targeting heparan sulfate and chondroitin sulfates of the cell surface glycocalyx. Chondroitin sulfate A targeting improved uptake in cells seeded on stiff biomimetic devices, which is attributed to increased chondroitin sulfate proteoglycan localization on cell surfaces in comparison to cells grown on soft devices. These results demonstrate the critical role that mechanical stiffness and stretch play in the alveolus and the importance of including these properties in nanotherapeutic design.

## Highlights

• Lung alveolus biomimetic devices have tunable stiffnesses for probing mechanical effects
• Gene expression across cancer relevant pathways are altered by interstitial stiffness
• Proteoglycan distribution is altered in cells grown on materials with different stiffnesses
• Nanoparticle uptake increases for cells on biomimetics by targeting of chondroitin sulfate

## Graphical abstract

**Figure:**
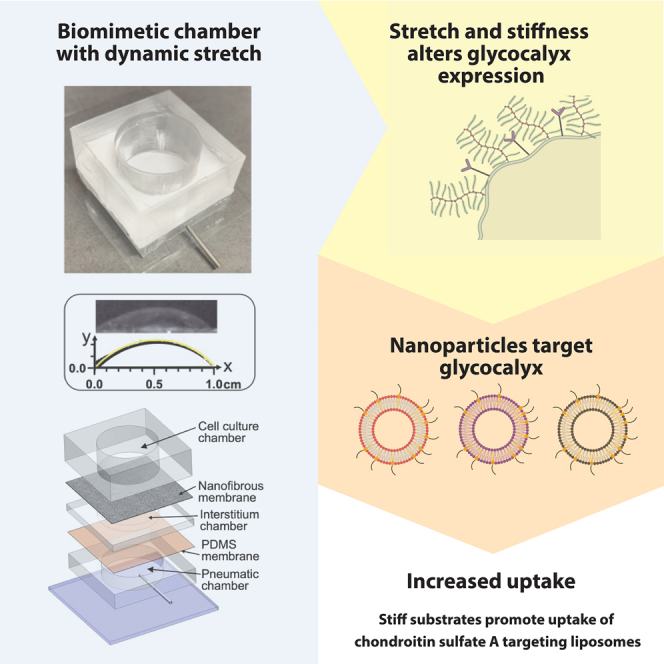

## Introduction

Lung cancer is the leading cause of cancer-related death worldwide, with a mortality rate of about 22% in 2023.^1^ The high mortality rate stems from a combination of limited screening,^2^ highly metastatic potential of cells,^3^^,^^4^ relapse after common chemotherapeutic treatments,^5^ and absence of efficient alternative treatment options. In addition to overall changes in cell behavior, changes in the mechanical properties are a hallmark that often accompanies lung cancer progression.^6^ Specifically, the enhanced deposition and activity of fibroblasts, fibrocytes,^7^^,^^8^^,^^9^^,^^10^ and mesenchymal stems cells^11^ in the tumor microenvironment causes stiffening of the interstitial ECM,^12^^,^^13^ on which epithelial cells reside. Changes in mechanical environments alter a number of different cell behaviors in lung cancer cells that include metabolic processes and immune responses.^14^^,^^15^^,^^16^^,^^17^

The contribution of mechanical dysfunction is further complicated by the fact that alveolar epithelial cells, a lineage source of many non-small cell lung cancers,^18^^,^^19^^,^^20^^,^^21^ are exposed to three-dimensional (3D) stretching cycles that coincide with breathing. Indeed, cyclic stretching applied to cells can change the tightness of junctions^22^ and alter the expression of genes in lung tissues,^23^ including lung cancer derived cell lines. The importance of cyclic stretch has led to the development of biomimetic devices that seek to include stretch in their models.^24^^,^^25^^,^^26^^,^^27^ While the effects of cyclic stretch are documented with accurate models for lung interfaces, the interplay between stretch and physiologically relevant interstitial stiffnesses is not accurately modeled in biomimetic models.

Although the contributions of ECM are well documented in cancer etiology,^28^^,^^29^^,^^30^ conventional culture models used to develop therapeutics do not effectively mimic this dynamic environment. Thus, they fail to reflect mechano-transmitted cues that play essential roles in many disease etiologies.^31^^,^^32^^,^^33^^,^^34^ This stiffening process is known to affect how nanomaterials interact with epithelial cells and lung fibroblasts as increased stiffness promotes uptake of Au nanoparticles (NPs) while reducing uptake of carbon nanotubes.^35^^,^^36^^,^^37^^,^^38^^,^^39^ However, designs that take advantage of substrate driven differences in cells have not been realized nor have the effects of dynamic forces been included in combination.

To assess how lung interstitial stiffness affects A549 lung cancer derived cells, a model system for lung cancer, we assessed the changes in gene expression in cells cultured on tissue culture plastic and biomimetic structures. Notably, our biomimetic structures have cyclic 3D stretch with tunable interstitial stiffness allowing us to mimic healthy and fibrotic/cancerous conditions. Using RNA sequencing, we identify upregulated and downregulated genes corresponding to pathways related to actin cytoskeleton, metabolism, and cancer associated proteoglycans that change in response to environmental cues. Based on these observations, we produce lipid NPs with specific glycocalyx recognition peptides on the surfaces focused on important lung cancer specific glycosaminoglycans (GAGs). We demonstrate that chondroitin sulfate A (CSA) targeting liposomes led to better uptake efficiency in A549 cells grown on a fibrotic, mechanically stretched environment, a result not observed in tissue culture plastic conditions. Taken together, this study demonstrates the value in considering environmental cues in the design of nanoparticle systems.

## Results and discussion

### Fabrication and characterization of dynamic alveolar interstitium platform

**Figure 1:**
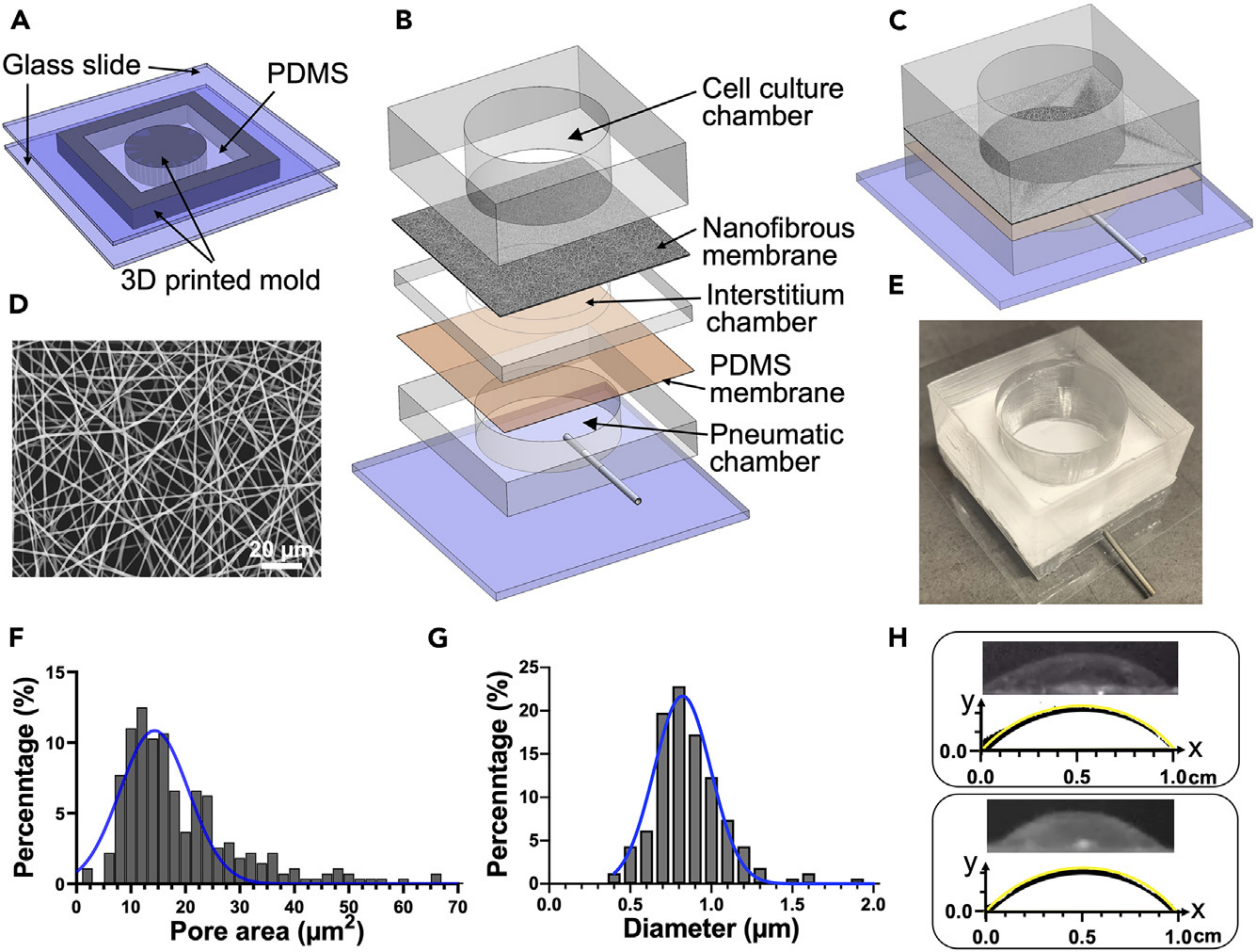
Design and characterization of the platform (A) Fabrication of the layer for cell culture, interstitium, and pneumatic chambers. The structures of the layers were the same with different heights (8mm for cell culture layer, 1mm for interstitium layer and 4mm for pneumatic layer). (B) Exploded illustration of the platform fabrication. (C) Illustration of the assembled platform. (D) SEM image of the nanofibrous membrane. (E) Image of the platform. (F) Pore size distribution and (G) fiber diameter distribution of the nanofibrous membrane. (H) Characterization of the 3-D mechanical stretch. Original CCD images and the profiles (black curve) of the deformed PDMS membrane bonded on the pneumatic chamber (upper frame) or nanofibrous membrane on the interstitium bonded on the pneumatic chamber (lower frame) under a theoretical strain of 15% (yellow curve). The scales of axis x and y were the same.

To understand how mechanical environment and dynamic forces impact cell interactions with NPs, we created a dynamic platform consisting of a cell culture chamber, an interstitium layer, and a pneumatic chamber (Figure 1), made of polydimethylsiloxane (PDMS). A biomimetic nanofibrous membrane was placed on top of the interstitium layer, which was sandwiched between the cell culture chamber and the pneumatic chamber (Figures 1B–1E). The assembly between these parts was completed by using a PDMS thin film adhesive as we previously reported (See the STAR Methods).^40^

The nanofibrous membrane was generated by electrospinning a polycaprolactone (PCL) solution to mimic the nanofibrous basement membrane of alveolar epithelium since the PCL nanofibrous membrane has been reported to support epithelium formation and favor cell-cell crosstalk.^41^^,^^42^^,^^43^ It is also an appropriate choice for this device due to its slower degradation rate, which allows for extended cell culture time. The nanofibrous membrane had an average diameter of 820 ± 180 nm and an average pore size of 14.3 ± 6.3 μm^2^ (Figures 1D, 1F, and 1G). The interstitium layer was prepared with PDMS, and its stiffness was determined by tailoring the type and composition of PDMS to replicate the stiffness of normal (1–5 kPa) and fibrotic (20–100 kPa) lung tissues,^44^^,^^45^^,^^46^ i.e., PDMS Sylgard 527 for 5 kPa and a 10:1 (w/w) mixture of Sylgard 527 and 184 for 50 kPa, respectively.^47^ The nanofibrous membrane was first oxygen plasma treated and coated with type I collagen, and then immobilized on the PDMS interstitium, thus allowing the cells to adhere to the nanofibers but not onto the PDMS upon seeding. As such, the nanofibers provided contact guidance for the cell to adhere and grow, while the interstitium only regulates the cells with its stiffness. Moreover, to imitate the breath movement, a theoretical 15% linear strain was applied at a frequency of 0.2 Hz. When air was infused into the pneumatic chamber, the experimental observations of the strains of the PDMS membrane and the nanofibrous membrane immobilized on the interstitium agreed with the theoretical calculation (Figure 1H), indicating that the 3-D mechanical stretch was transmitted through the interstitium to the nanofibrous membrane, which provided mechanical stimulation to the adherent cells. Taken together, this platform provides a biomimetic system for evaluating nanoparticle targeting.

### Lung epithelium gene expression changes in response to mechanical environment

**Figure 2:**
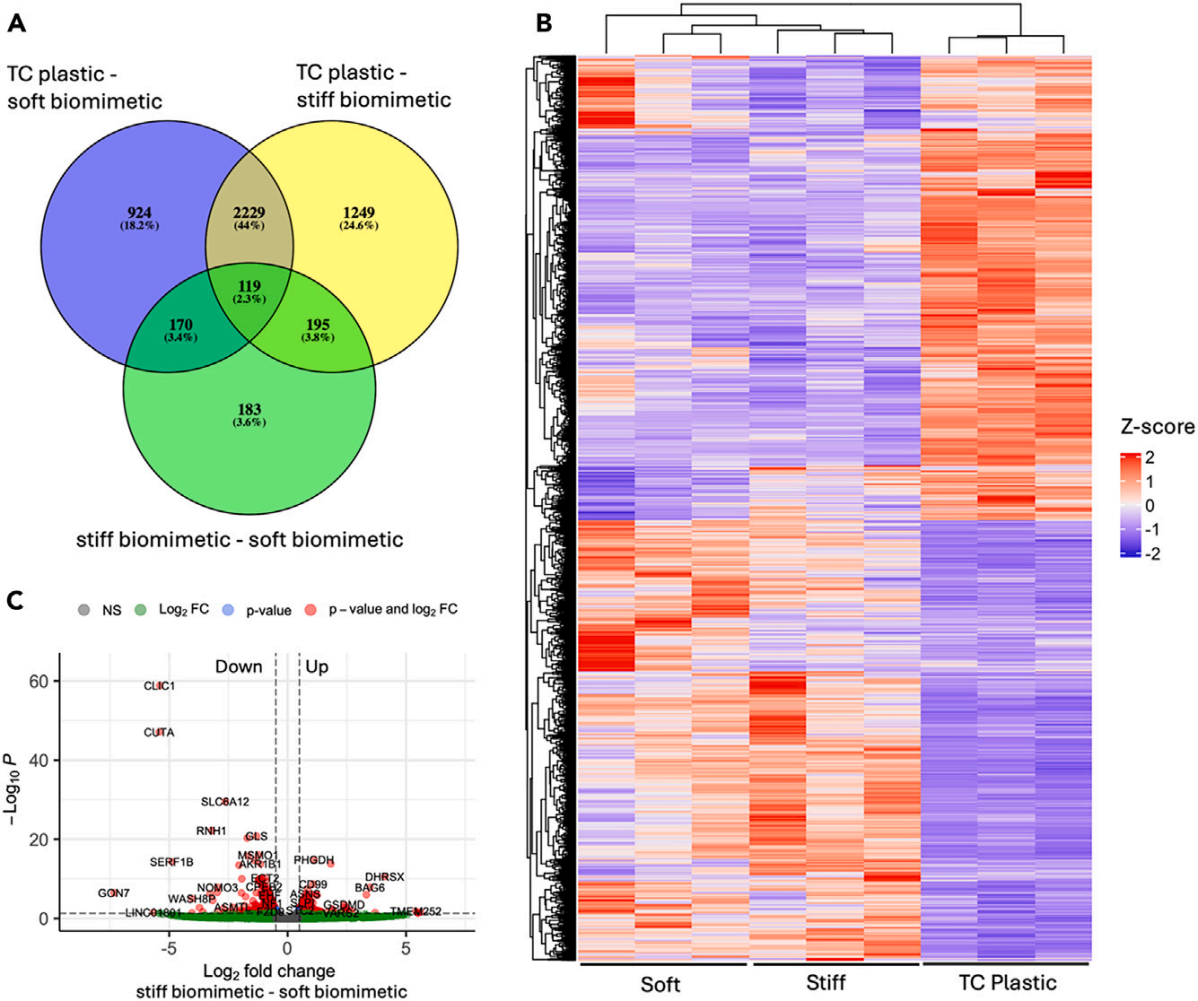
Transcriptomic analysis of A549 cells grown under different conditions (A) Venn diagram showing the number of DEGs comparing all conditions. (B) Heatmap of all DEGs across all conditions that have been clustered. (C) Volcano plot of the DEGs between soft and stiff interstitium.

To profile how changes in mechanical environment alter gene expression, we performed RNA sequencing of enriched mRNA collected from A549 cells grown on biomimetic chips, soft and stiff, along with those grown on conventional tissue culture plastic controls. After sequencing, we identified differentially expressed genes (DEGs; log_2_ fold change >0.5 and *p* < 0.05; Figure 2). From this analysis, we found over 5000 genes that were differentially expressed across all conditions (Figure 2A). Given the number of DEGs across the different conditions, we performed a combination of gene ontology (GO) and KEGG pathway analysis to identify biological processes and pathways that have been upregulated and downregulated depending on the A549 cellular environment.

Notably, when we examine DEGs that are regulated regardless of interstitial stiffness when cyclic stress is applied (overlap between yellow and purple circles; Figure 2A), we observe that many of the genes involved in metabolic processes are upregulated by TC plastic (Tables 1, 3, S1, S2, S3, S4, S5, S6, S7, and S8). This is consistent with observations that high stiffness substantially alters metabolic pathways in lung cancer because the TC plastic is several orders of magnitude stiffer than our biomimetic devices.^16^^,^^17^ However, biomimetic devices promote expression of genes in several key GOs related to cell junctional organization (specifically epithelial) and actin cytoskeletal organization. Cell junctional organization promotion by cyclic stretch is consistent with our observations along with others that tight junction formation increases in A549 cells grown on biomimetic structures with 3D cyclic stretch.^48^^,^^49^ Furthermore, KEGG analysis points to HIF1 activation pathways when we apply cyclic stretch regardless of stiffness, a canonical response of cyclic stretch in A549 cells (Table 3).^50^^,^^51^ Overall, these observations point to tighter junction formation and activation of pathways consistent with stiffness and stretch that overall provide evidence that our biomimetic cyclic stretch aligns with literature precedents regardless of the underlying stiffness of the interstitial layer in our devices.

**Table 1:** Upregulated gene ontology

Sample	Biological Process	ID	*p*-value
TC Plastic – Overlap of Soft/Stiff Biomimetic	ncRNA metabolic process	GO:0034660	1.94E-11
	cell cycle process	GO:0022402	1.92E-10
	mitochondrial gene expression	GO:0140053	6.21E-10
	organonitrogen compound metabolic process	GO:1901564	1.44E-09
	small molecule metabolic process	GO:0044281	7.57E-09
	DNA metabolic process	GO:0006259	2.87E-08
	mitochondrion organization	GO:0007005	1.0763E-05
	electron transport chain	GO:0022900	1.1621E-05
	cellular lipid metabolic process	GO:0044255	0.00027136
	cellular response to stress	GO:0033554	0.00049641
	organic substance catabolic process	GO:1901575	0.00316609
	carbohydrate derivative metabolic process	GO:1901135	0.0040428
	fibroblast proliferation	GO:0048144	0.00462458
	mitochondrial RNA metabolic process	GO:0000959	0.00604469
	[2Fe-2S] cluster assembly	GO:0044571	0.00761101
	mitochondrial potassium ion transmembrane transport	GO:0140141	0.01004394
	methylation	GO:0032259	0.01014039
	homeostatic process	GO:0042592	0.01080513
	positive regulation of biological process	GO:0048518	0.01940453
	terpenoid metabolic process	GO:0006721	0.02014444
	nuclear DNA replication	GO:0033260	0.02469075
	response to xenobiotic stimulus	GO:0009410	0.03164244
	regulation of cellular component organization	GO:0051128	0.03811142
TC Plastic – Soft Biomimetic	DNA-templated DNA replication	GO:0006261	1.35E-13
	cell cycle	GO:0007049	7.20E-10
	DNA duplex unwinding	GO:0032508	7.1976E-06
	cell division	GO:0051301	9.6165E-05
	organelle fission	GO:0048285	0.00226783
	proton transmembrane transport	GO:1902600	0.00459343
	alcohol biosynthetic process	GO:0046165	0.02198829
	G2/M transition of mitotic cell cycle	GO:0000086	0.02750371
	cellular respiration	GO:0045333	0.03969075
	DNA replication checkpoint signaling	GO:0000076	0.04585374
TC Plastic – Stiff Biomimetic	ribosome biogenesis	GO:0042254	6.13E-18
	organonitrogen compound metabolic process	GO:1901564	1.94E-13
	mitochondrion organization	GO:0007005	7.22E-10
	respiratory electron transport chain	GO:0022904	3.82E-09
	RNA modification	GO:0009451	0.00028212
	regulation of intrinsic apoptotic signaling pathway	GO:2001242	0.00130154
	cellular response to stress	GO:0033554	0.00221124
	RNA localization	GO:0006403	0.00435245
	U2-type prespliceosome assembly	GO:1903241	0.00579257
	mitotic metaphase chromosome alignment	GO:0007080	0.01227046
	cytochrome complex assembly	GO:0017004	0.02895312
	tRNA metabolic process	GO:0006399	0.03220472
Stiff Biomimetic – Soft Biomimetic	amide metabolic process	GO:0043603	8.76087348
	amino acid metabolic process	GO:0006520	6.59125767
	L-serine metabolic process	GO:0006563	2.53890432

While cyclic stretch effects on A549 cells are well established, the interplay between interstitial stiffness and cyclic stretch is poorly understood. Therefore, we examined how stiffness in the presence of cyclic stretch alters cell response in comparison to TC plastic conditions, an important topic due to the underlying mechanical nature of lung cancers. Interestingly, we do observe upregulation of epithelial to mesenchymal transition (EMT) DEGs in cells grown on soft biomimetics compared to TC plastic (purple only portion of circle Figure 2A), an important observation in the context of lung cancer metastatic modeling where mechanical regulation shares similar pathways to EMT (Figure S1; subset of clustered results in Table 2). When we evaluate DEGs related to stiffness (i.e., soft, healthy interstitium compared to stiff interstitium similar to fibrotic conditions; entire green circle, Figure 2A) on our biomimetic models, we observe 3 key changes: (1) GO biological processes related to epithelial migration are expressed more highly in cells that are grown on soft biomimetic substrates (Table 2). This observation, in conjunction with our other observations that soft interstitium promoted epithelial to mesenchymal transitions, points to a more dysregulated and mobile epithelial phenotype in the A549 cells grown on soft biomimetics with potential implications in metastatic modeling (although outside the scope of this project). (2) Stiff interstitium promotes the expression of genes related to amino acid metabolic pathways. This aligns with stiffness changes in metabolic processes observed in native lung cancer cells, again highlighting an important distinction in our biomimetic system (Tables 1 and 3). (3) Soft interstitium promotes upregulation of proteoglycans related to cancer in comparison to stiff interstitium (Table 3). This KEGG pathway is linked to hyaluronan, chondroitin sulfates, and heparan sulfates along with various proteins. Glycan and glycocalyx changes are a hallmark of many cancers, including lung cancers. In the case of lung cancers, the types of glycosaminoglycans (GAGs), especially chondroitin and heparan sulfates, change depending on the cancer source.^52^^,^^53^ Specifically, chondroitin sulfates are upregulated in comparison to heparan sulfates in non-small lung cancer cells compared to nearby healthy tissue.^53^ Our model suggests that stiffness plays a role in this process. Based on this observation, we sought to evaluate how GAG localization changes in the cells on the biomimetic substrates and develop NPs targeting different GAGs to evaluate how stiffness in biomimetic models alters potential recognition.

**Table 2:** Downregulated gene ontology (also see Figure S1)

*Sample*	*Biological Process*	*ID*	*p-value*
TC Plastic – Overlap of Soft/Stiff Biomimetic	anatomical structure development	GO:0048856	2.83E-26
	small molecule metabolic process	GO:0044281	2.5644E-06
	cell junction organization	GO:0034330	7.677E-06
	cytoskeleton organization	GO:0007010	2.2855E-05
	extracellular matrix organization	GO:0030198	0.0006875
	regulation of primary metabolic process	GO:0080090	0.00190789
	complement activation	GO:0006956	0.00206795
	regulation of nitrogen compound metabolic process	GO:0051171	0.0094928
	negative regulation of molecular function	GO:0044092	0.01230769
	organonitrogen compound metabolic process	GO:1901564	0.02170224
	cytolysis by host of symbiont cells	GO:0051838	0.02318679
	negative regulation of phospholipid biosynthetic process	GO:0071072	0.04930436
TC Plastic – Soft Biomimetic	anatomical structure morphogenesis	GO:0009653	3.2806E-06
	cell projection organization	GO:0030030	0.01235392
	negative regulation of cell population proliferation	GO:0008285	0.03754319
TC Plastic – Stiff Biomimetic	regulation of RNA biosynthetic process	GO:2001141	1.44E-12
	anatomical structure morphogenesis	GO:0009653	7.579E-06
	cellular response to endogenous stimulus	GO:0071495	0.00558597
Stiff Biomimetic – Soft Biomimetic	cell cycle	GO:0007049	5.75E-08
	establishment of localization in cell	GO:0051649	6.6261E-06
	epithelial cell migration	GO:0010631	0.00029803
	macromolecule modification	GO:0043412	0.00035592
	anatomical structure development	GO:0048856	0.00087536
	intracellular signal transduction	GO:0035556	0.00662923
	homeostatic process	GO:0042592	0.01954022
	cell division	GO:0051301	0.02188047
	protein localization to kinetochore	GO:0034501	0.02199073
	cellular response to external stimulus	GO:0071496	0.02691698
	growth	GO:0040007	0.0424402
	cellular response to oxygen-containing compound	GO:1901701	0.0428945

**Table 3:** KEGG pathway analysis

Sample	KEGG Pathway	KEGG ID	Count	Regulation Direction
TC Plastic – Overlap of Soft/Stiff Biomimetic	Aminoacyl-tRNA biosynthesis	KEGG:00970	14	Upregulated
	Metabolic pathways	KEGG:01100	134	Upregulated
	p53 signaling pathway	KEGG:04115	16	Upregulated
	Cell cycle	KEGG:04110	24	Upregulated
	Complement and coagulation cascades	KEGG:04610	26	Downregulated
	Glycolysis/Gluconeogenesis	KEGG:00010	18	Downregulated
	HIF-1 signaling pathway	KEGG:04066	20	Downregulated
	Biosynthesis of amino acids	KEGG:01230	14	Downregulated
	Central carbon metabolism in cancer	KEGG:05230	13	Downregulated
	Carbon metabolism	KEGG:01200	17	Downregulated
	Fructose and mannose metabolism	KEGG:00051	8	Downregulated
TC Plastic – Soft Biomimetic	Fanconi anemia pathway	KEGG:03460	8	Upregulated
	Steroid biosynthesis	KEGG:00100	5	Upregulated
	Cell cycle	KEGG:04110	13	Upregulated
	Oxidative phosphorylation	KEGG:00190	11	Upregulated
TC Plastic – Stiff Biomimetic	Parkinson disease	KEGG:05012	38	Upregulated
	Prion disease	KEGG:05020	37	Upregulated
	Amyotrophic lateral sclerosis	KEGG:05014	40	Upregulated
	Huntington disease	KEGG:05016	36	Upregulated
	Oxidative phosphorylation	KEGG:00190	23	Upregulated
	Pathways of neurodegeneration - multiple diseases	KEGG:05022	46	Upregulated
	Thermogenesis	KEGG:04714	30	Upregulated
	Proteasome	KEGG:03050	13	Upregulated
	Alzheimer disease	KEGG:05010	38	Upregulated
	Non-alcoholic fatty liver disease	KEGG:04932	19	Upregulated
	Diabetic cardiomyopathy	KEGG:05415	22	Upregulated
	Metabolic pathways	KEGG:01100	87	Upregulated
	Chemical carcinogenesis - reactive oxygen species	KEGG:05208	22	Upregulated
	Spinocerebellar ataxia	KEGG:05017	15	Upregulated
	Spliceosome	KEGG:03040	17	Upregulated
	Herpes simplex virus 1 infection	KEGG:05168	33	Downregulated
	Transcriptional misregulation in cancer	KEGG:05202	16	Downregulated
Stiff Biomimetic – Soft Biomimetic	Biosynthesis of amino acids	KEGG:01230	7	Upregulated
	Glycine, serine and threonine metabolism	KEGG:00260	5	Upregulated
	Ribosome	KEGG:03010	8	Upregulated
	Tyrosine metabolism	KEGG:00350	4	Upregulated
	Regulation of actin cytoskeleton	KEGG:04810	16	Downregulated
	Proteoglycans in cancer	KEGG:05205	15	Downregulated

### Lung epithelium glycosaminoglycan localization in response to mechanical environment

**Figure 3:**
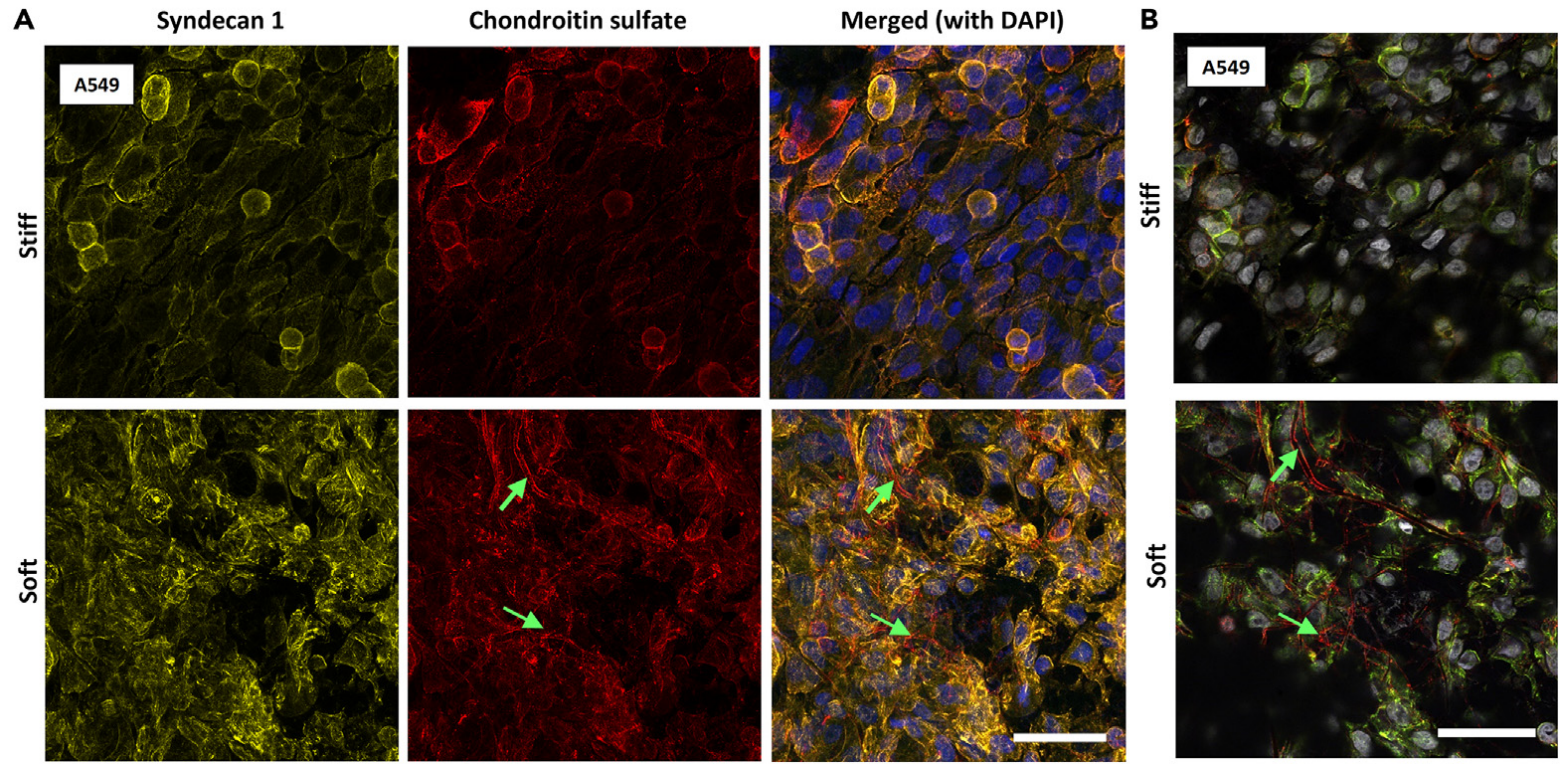
Effect of substrate mechanics on glycocalyx proteoglycan and glycosaminoglycan localization Immunofluorescent maximum intensity projections of epithelial cells for (A) syndecan-1 (yellow), chondroitin sulfate (red) with DAPI (blue) as a counterstain, cultured in soft and stiff matrix with 3-Dimensional stretch. (B) Syndecan-1 (yellow), chondroitin sulfate (red) localization around cell (nucleus stained with DAPI (gray)) shows a slice from the z stack showing chondroitin surface localization with fibers. Green arrows mark the chondroitin sulfate fibers that are delocalized from syndecan 1. Scale bar 100 μm. (also see Figures S2 and S3).

To have an in-depth understanding of sulfated glycosaminoglycans (GAGs) and their response to mechanical cues, we examined chondroitin sulfate and heparan sulfate localization changes on our biomimetic models and TC plastic. To this end, A549 cells were cultured on tissue culture plastic and biomimetic chips under dynamic conditions until they reached confluency. After fixing and staining with antibodies against chondroitin sulfate and heparan sulfate GAGs as well as syndecan-1 proteoglycans, the most highly expressed cell surface-linked proteoglycan, their localization was observed. Confocal micrographs show highly localized chondroitin sulfate around the cells, which also colocalized with syndecan-1 in stiff biomimetics (Figure 3). In contrast, soft matrices induced chondroitin sulfate localization within the fibrous structure of the nanofibrous basement membrane and localization with the cell surface. Notably, fibrous chondroitin sulfate structures that are not colocalized to the transmembrane, syndecan 1, are present (Figure 3). Similar experiments with heparan sulfate showed no definitive change in stiffness-induced localization (Figure S2). In contrast, cells grown in tissue culture plastic exhibited lower expression of chondroitin sulfate and heparan sulfate proteoglycans on the cells, with slight colocalization with syndecan-1 around the cells (Figure S3). This shows that cells grown in tissue culture plastic have differential localization GAGs production of epithelial cells in comparison to these biomimetics. Collectively, these results show that chondroitin sulfate localization is dependent on the membrane stiffness when dynamic stretches are applied.

### Designing peptide functionalized liposomes targeting cancer associated Glycans

Based on our observations that proteoglycan expression and GAG localization changes as a function of dynamic stretch and stiffness of the supporting substrate, we designed liposomes aimed at facilitating uptake into cells depending on the mechanical environment. Since we observed differences in the expression of different types of glycoproteins, we sought to create liposomes that recognize specific GAGs. Known peptide structures that interact with chondroitin sulfate A (CSA), chondroitin sulfate C (CSC), or heparan sulfates (HS; see Table 4 for sequences) were utilized for coating the liposomes. We note that these peptides (and related ones) often have cross-reactivity with other GAGs but have chosen ones with higher affinity for each target.^54^^,^^55^^,^^56^ For these studies, we modified the peptides to include cysteines to allow easy functionalization using bioconjugation strategies. To facilitate coating of the liposome surface, peptide-lipid conjugates were synthesized by reacting to a maleimide-terminated polyethylene glycol (PEG; 20 kDa) lipid with our cysteine-terminated thiols in anhydrous conditions (Figure S4). Peptide conjugates were subsequently purified using HPLC. Synthesis of conjugates was confirmed by comparing them with two different controls. Firstly, peptide only controls and peptide-lipid conjugates were run in a polyacrylamide gel followed by Coomassie staining. The results show an upward shift of bands in conjugates compared to the peptides, which confirms the size shift as conjugates run more slowly than peptide only controls in the gel (Figure S5A). Secondly, to confirm that the detected bands in the conjugate lanes in the Coomassie gel had peptides, we performed a labeling assay in a two-step process. Amines on the peptides were labeled with methyltetrazine by incubating conjugates with an N-Hydroxysuccinimide ester (NHS) methyltetrazine. Second, a *trans*-cyclooctene fluorophore was reacted with peptides, and the product was run in a polyacrylamide gel. As a control, lipids without peptides were treated in the same way, and no conjugation was observed (Figure S5B). These results indicate that we successfully synthesized the lipid-peptide conjugates.

**Table 4:** Peptides used for targeting with primary recognition GAGs

Peptide Sequence	Primary Recognition GAG	Net charge (pH 7.4)
CERRIWFPYRRF	Chondroitin sulfate C Butterfield et al.^57^	1.89
CRTPPESYASVR	Chondroitin sulfate A Loers et al.^54^	0.89
CGRKKRRQRRRPQ	Heparan sulfates Tyagi et al.^58^	7.89

**Figure 4:**
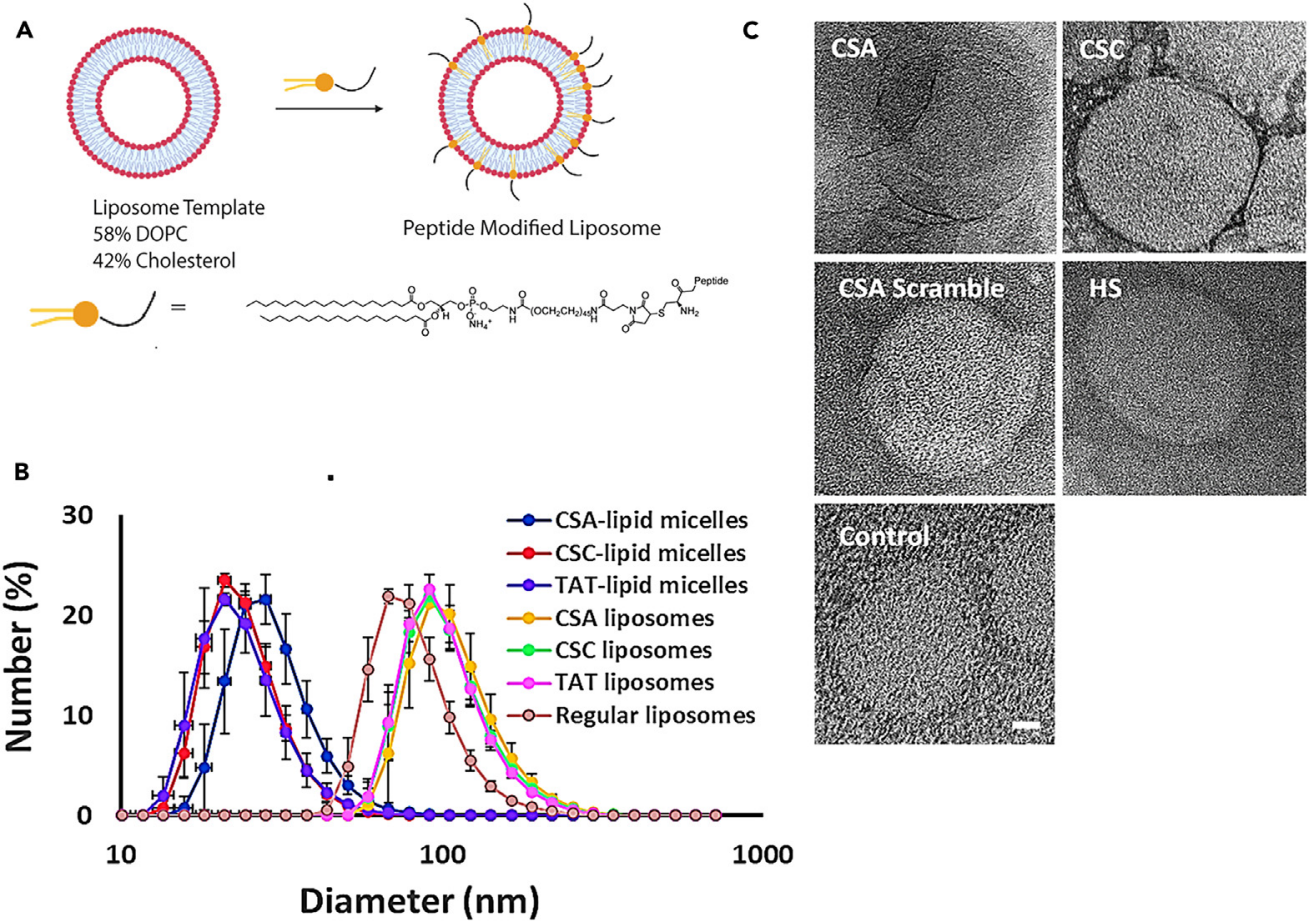
Design of nanoparticles for targeting glycocalyx changes (A) Schematic depicting the strategy for modifying liposomes with peptides that target different glycocalyx GAGs. (B) Dynamic light scattering size distributions for peptide lipid micelles of chondroitin sulfate A (blue), chondroitin sulfate C (red), heparan sulfate (purple), bare liposomes (Regular, orange) along with peptides targeting chondroitin sulfate A (CSA, yellow), chondroitin sulfate C (CSC, green), and heparan sulfate (HS, pink). (C) Transmission electron microscopy (TEM) images of liposomes modified with targeting peptides chondroitin sulfate A (CSA), chondroitin sulfate C (CSC), heparan sulfate (HS) and chondroitin sulfate A scramble (CSA Scramble) and bare liposomes (control). Scale bar = 20 nm; applies to all images. (also see Figures S5–S8; Tables S9 and S10).

Next, we created base liposome structures consisting of 58 mol % 1,2-dioleoyl-*sn*-glycero-3-phosphocholine (DOPC) and 42 mol % cholesterol that were systematically extruded to 100-nm diameters. The lipid-peptide conjugates, which formed small micelles, were then incubated with the pre-formed liposomes to allow for spontaneous intercalation into the liposome (Figure 4A). Dynamic Light Scattering (DLS) also shows the micelles of the lipid peptide conjugates and that there is an increase in apparent liposome diameter upon incubation with preformed liposomes (Figures 4C; Table S9) that is consistent with expected size shifts for our peptide-conjugated liposomes. To confirm that the morphology of the particles does not change, we examined the liposomes with transmission electron microscopy (TEM). No significant morphological changes were observed between base and modified liposomes (Figure 4B), while all the liposomal formulations displayed similar size distributions (Figure S6). The size distribution obtained from DLS also aligns well with our TEM images as peptide attached liposomes appeared larger than control liposomes. As an aside, uranyl acetate staining gave better contrast for all modified liposomes compared to unmodified, further highlighting changes to the exterior structure. To further confirm that peptides are indeed attached to the liposomes we performed a peptide quantification assay. Our results show that around 200–400 ligands are attached to each liposome surface (Figure S7). Bare liposomes served as a baseline control for the calculations of modified liposomes.

In addition, we evaluated the charge of our liposomes before and after peptide modification. Overall, the zeta potential average values were fairly neutral in all liposome formulations (Table S10). The change in mean values is negligible between different formulations, which is likely due to the broader distribution spectrum observed in smaller-sized liposomes.^59^ However, a small positive shift in the distribution curve was observed in peptide modified liposomes compared to the bare control liposomes (Figure S8), which can be attributed to the charged peptides on the surface (Table 4). Taken together, all these liposome formulations are nearly neutral in PBS, consistent with DOPC liposomes.

### Effect of mechanical environment on nanoparticle uptake

**Figure 5:**
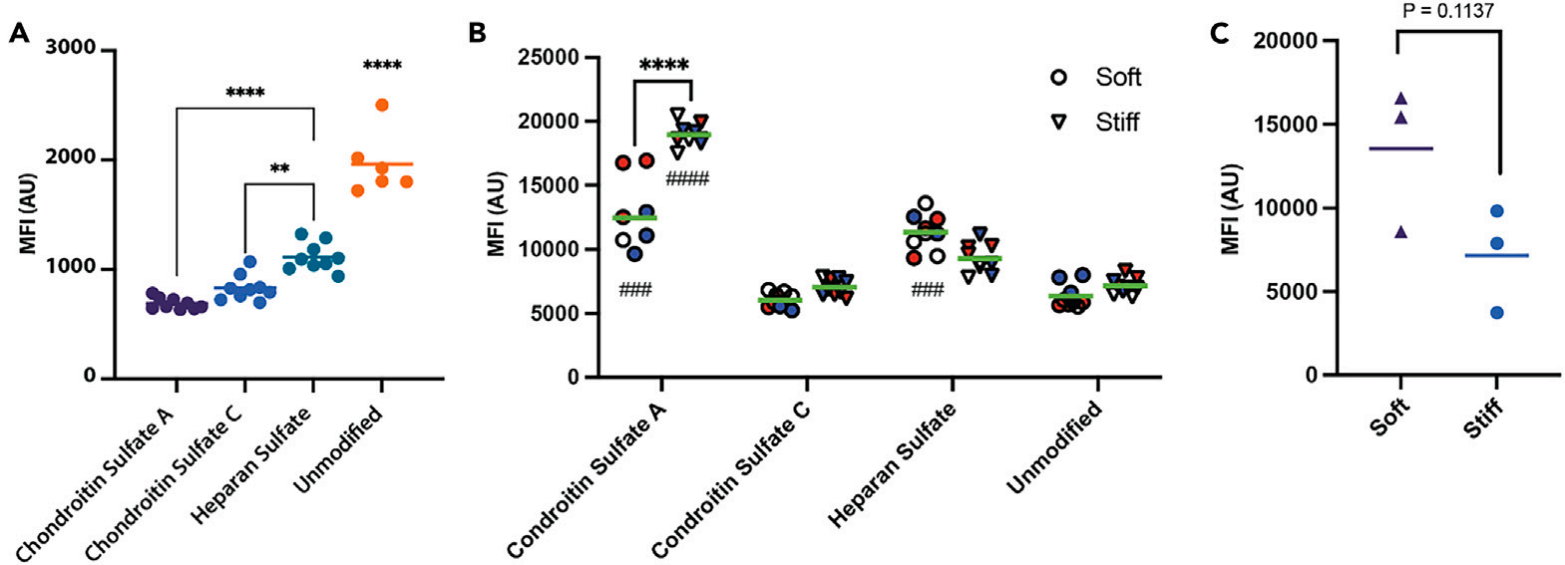
Uptake of liposomes into A549 cells in different mechanical environments (A–C) Flow cytometric median fluorescence intensities of A549 cells grown TC Plastic (A), on soft or stiff substrates undergoing dynamic cyclic stretches (B), with CSA scramble control liposome on soft or stiff substrates undergoing dynamic cyclic stretches (C), following treatment with fluorophore labeled liposomes formulated with different targeting peptides. ∗∗∗∗*p* < 0.0001, ∗∗∗*p* < 0.001, ∗∗*p* < 0.01, and ∗*p* < 0.05; two-way ANOVA with Tukey’s HSD post-hoc analysis for comparing for substrate effects and relative to control liposome ####*p* < 0.0001, ###*p* < 0.001 in comparison to unmodified liposomes. Different colors come from biological replicates.

Next, we assessed whether there were differences in liposome uptake depending on the surface peptide and the culture conditions. For these studies, the liposomes were formulated with lipophilic fluorophores to allow assessment with flow cytometry. First, we assessed liposome uptake in conventional screening conditions (TC plastic). Liposomes were incubated with A549 cells, and uptake was assessed via flow cytometry. In TC plastic conditions, unmodified liposome controls show greater uptake than all targeting structures (Figure 5A). HS liposomes enhanced uptake compared to the liposomes functionalized with CSC and CSA peptides (Figure 5A). Overall, this aligns with our observation that the HS and CS glycocalyx are only weakly present on cells grown on TC plastic (Figure S3).

To assess whether the mechanical environment altered liposome uptake, A549 cells were cultured on the alveolar interstitium platforms for 1 week to allow the cells to reach monolayer confluency. Again, we utilized soft (5 kPa) and stiff (50 kPa) conditions with dynamic stretch cultures to assess how the environment affected nanoparticle uptake. When a dynamic stretch was introduced to the A549 cells, CSA-targeting liposomes showed enhanced uptake into cells grown on stiff substrates compared to the controls (unmodified liposomes; Figure 5B). Furthermore, targeting CSA and HS enhanced uptake compared to unmodified liposomes in contrast to our TC plastic studies. To ensure that this process was not charge driven, we used a scramble peptide sequence for CSA, which then resulted in no significant difference in uptake between soft and stiff substrates (Figure 5C). These results conclusively show that mechanical stiffness changes the uptake preference of different liposome formulations.

Changes in the localization of GAGs around the cells in our biomimetic models explain higher chondroitin sulfate targeting in stiff matrix compared to dynamic soft conditions. The strict colocalization of chondroitin sulfate GAGs with the cells in stiffer matrix contributes to the increased uptake of CSA targeting liposomes. Similarly, lack of GAGs limits entry for targeted liposomes in all TC plastic conditions. On the contrary, we see that the upregulation of GAGs present in all our biomimetic models compared to conventional TC plastic can also have a mixed effect. However, targeting tissues based on GAGs is a complex process. High levels of HS on cells grown in biomimetics do not necessarily improve entry for HS targeting liposomes. Others have observed that GAG content is both necessary for entry for the peptide we used and potentially inhibitory at high levels.^58^^,^^60^^,^^61^^,^^62^ These observations, along with our results, point to the need to explore multiple combinations of targeting as we have here to identify high-performing formulations.

### Conclusion

Alveolar epithelial cells are subjected to constant cyclic stretches and frequently encounter stiffer ECM in a fibrotic or cancerous lung. We have devised a cell culture platform that mimics these dynamic and mechanical characteristics of ECM to study lung alveolar cells *in vitro*. Our studies demonstrate stiffness plays a significant role across a host of different pathways that differ significantly from conventional culture conditions, with many cancer-specific pathways differentially regulated. These culture environment conditions can alter how nanomaterials target cells and the design of potential therapeutics. Future studies could focus on potential material alternatives for the device’s critical components through integration of native interstitial proteins. Looking forward, it will be important to contextualize variations to clinical samples and compare them to potential animal models. Such efforts are especially critical given the fact that biomimetic models are poised to play an essential role in future therapeutic discovery given the limitations of animal models in replicating human disease and predicting efficacy.

### Limitations of the study

A549 cancer cell lines will have differences compared to patient derived primary cancer cells that can alter nanoparticle interactions. Significant heterogeneity, including spatial, cell types, and mechanical, exists within tumor environments that are not included in these models that can further modulate cell behaviors.

## Resource availability

### Lead contact

Requests for further information, resources, or materials should be directed to and will be fulfilled by the lead contact, B.M. (brian.meckes@unt.edu), upon reasonable request.

### Materials availability

No new unique materials were generated by this study.

### Data and code availability

• Data: Processed RNA-seq data is available through the gene expression omnibus (GSE272292).
• Code: This paper does not include original code.
• All other requests: Any additional information or data included in this manuscript and the supplemental material will be made available by the lead contact upon reasonable request.

## Acknowledgments

Research reported in this publication was supported by the University of North Texas Seed Funds (B.M. and Y.Y), and the 10.13039/100000002National Institutes of Health under awards R35GM150577 (B.M.), and R15EY033967 (Y.Y). The authors would also like to thank Camrie Johnson, Olivia Wang, and Sachin Kaluarachchi for their valuable contribution to this research. The authors would also like to express their gratitude to Ethan Ozment and Phoebe Doss from EMCore at the University of Texas Southwestern, as well as Giacomo Spano from University of North Texas Health Science Center, for their valuable guidance during the experiments conducted at their facility.

## Author contributions

Conceptualization, A.I.K., Ku.M., A.H., Y.Y., and B.M.; Methodology, A.I.K., Ku.M., A.H., Y.Y., and B.M.; Investigation, A.I.K., Ku.M., A.H., Ka.M., J.W., J.F., and P.R.; Writing – Original Draft, A.I.K., Ku.M., A.H., Y.Y., and B.M.; Writing – Review and Editing, A.I.K., Ku.M., A.H., Ka.M., J.W., J.F., P.R., Y.Y., and B.M.; Funding Acquisition, Y.Y. and B.M.; Resources, Y.Y. and B.M; Supervision, Y.Y. and B.M.

## Declaration of interests

The authors declare no competing financial interest.

## STAR★Methods

### Key resources table

**Table:** 

REAGENT or RESOURCE	SOURCE	IDENTIFIER
**Antibodies**		

Chondroitin Sulfate Monoclonal Antibody (CS-56)	Thermo Fisher Scientific	Cat# MA1-83055; RRID: AB_929919
Recombinant Anti-Syndecan-1 antibody [EPR6454]	Abcam	Cat# ab128936; RRID: AB_11150990
Ab-Heparan Sulfate (JM-403)	AMSBIO	Cat# 370730-1; RRID: AB_10890960
Goat anti-Rabbit IgG (H + L) Highly Cross-Adsorbed Secondary Antibody, Alexa Fluor™ 546	Thermo Fisher Scientific	Cat# A-11035; RRID: AB_2534093
Alexa fluor 594TM goat anti-mouse IgM μchain	Thermo Fisher Scientific	Cat# A-21044; RRID: AB_2535713

**Chemicals, peptides, and recombinant proteins**		

CERRIWFPYRRF	Genscript	N/A
CRTPPESYASVR	Genscript	N/A
CGRKKRRQRRRPQ	Genscript	N/A

**Critical commercial assays**		

Invitrogen™ PureLink™ RNA Mini Kit	Thermo Fisher Scientific	Cat# 12-183-018A
NextSeq 75bp High-Output kit	Illumina	Cat# 20024906
Pierce™ BCA Protein Assay Kit	Thermo Fisher Scientific	Cat# 23227
Pierce Quantitative Fluorometric Peptide Assay Kit	Thermo Fisher Scientific	Cat# 23290
Phosphatidylcholine Assay Kit	Abcam	Cat# ab83377

**Deposited data**		

RNA Sequencing Data	GEO	GSE272292

**Experimental models: Cell lines**		

Human alveolar epithelial cells (A549)	ATCC	Cat#: CCL-185 RRID:CVCL_0023

**Software and algorithms**		

ImageJ	Schindelin et al.^63^	http://rsb.info.nih.gov/ij/index.html
Galaxy Project	Galaxy Project Team	https://galaxyproject.org
g:Profiler	Kolberg et al.^64^	https://biit.cs.ut.ee/gprofiler/gost
Graphpad Prism 8/Prism 9	Graphpad, Inc	https://www.graphpad.com
R V4.4.1	R Project	https://www.r-project.org

### Experimental model and study participants

#### Cell line

Human alveolar epithelial cells (A549; Cat#: CCL-185, ATCC, male) were cultured in Dulbecco’s Modified Eagle Medium (DMEM) with L-glutamine (Life Technologies) supplemented with 10% fetal bovine serum (FBS), 100 U/ml penicillin and 100 μg/mL streptomycin (Life Technologies). Cells were cultured in the incubator of 37°C and 5% CO_2_. 4′,6-Diamidino-2-phenylindol (DAPI) stains did not show signs of mycoplasma.

### Method details

#### Fabrication of alveolar interstitium

All the parts of the platform except the nanofibrous membrane were made by casting the mixture of PDMS (Sylgard 184, Dow Corning) resin and curing agent at a 10:1.05 ratio (w/w) on the 3-D printed molds, followed by a curing process at 75°C for 2 h. Meanwhile, a thin PDMS membrane was prepared via spin-coating at 2500 rpm for a minute on a silicon wafer and curing at 75°C for an hour, and then bonded onto the pneumatic layer to construct the pneumatic chamber using a microtransfer assembly (μTA) technique that we previously developed.^40^ Briefly, the pneumatic layer with the opening facing down was stamped on a PDMS adhesive layer, which was prepared by spin-coating a PDMS mixture on a silicon wafer at 4000 rpm for 30 s, and transferred onto the PDMS membrane, followed by curing at 75°C for 1 h under a compressive pressure of approximately 1 MPa. The pneumatic layer with the bonded membrane was then gently peeled off from the silicon wafer. The middle interstitium layer was prepared by first bonding the interstitium layer onto the pneumatic layer with PDMS membrane using μTA technique and then adding Sylgard 527 (corresponding to 5 kPa normal lung tissue) or a 10:1 mixture of Sylgard 527 and 184 (50 kPa fibrotic tissue) into the interstitium chamber followed by curing at 75°C for 4 h.

Prior to cell culture, the electrospun nanofibrous membrane was bonded to the cell culture layer using the μTA technique, treated with oxygen plasma at medium power setting for 1 min in a plasma cleaner (Model PDC-001, Harrick Plasma), and bonded onto the interstitium layer. Subsequently, phosphate buffered saline (PBS) (BioWhittaker, Lonza) was added to the cell culture chamber to keep the fibrous membrane immersed and the whole platform was cured at 45°C overnight.

#### Electrospinning and characterization of nanofibrous membranes

A solution of PCL in 1,1,1,3,3,3-hexafluoro-2-propanol (HFIP, 10%, w/v) was electrospun to produce the nanofibrous membrane. Briefly, the PCL solution was loaded into a syringe with a gauge blunt-tipped needle as the spinneret and ejected using a syringe pump (New Era Pump Systems Inc) at a flow rate of 0.5 mL/h. A square glass plate (4″ x 4″) was placed on an aluminum tap covered plate served as the fiber collector, which was displaced 16 cm away from the spinneret and grounded to the power supply. A voltage of 14 kV was applied to the spinneret using a DC power supply (Spellman Bertan) for 20 min.

The nanofibrous membrane was sputter-coated by gold (Denton Vacuum LLC) and images were taken using a scanning electron microscope (SEM, TM3030 Plus, Hitachi High-Technologies Co., Tokyo, Japan). The pore size and fiber diameter were analyzed using ImageJ. Briefly, the images were adjusted using threshold command. The area of each pore was analyzed using the function of analyze particles. The diameter of fibers was measured by drawing a line across the fiber and measuring the length. Then, a histogram line graph was generated using Prism 8 (GraphPad software) to show the distribution of the measured pore areas or fiber diameter.

#### Characterization of mechanical stretching

The PDMS membrane bonded on the pneumatic chamber or the platform without the cell culture chamber were used for the mechanical stretch characterization. When air was infused into and withdrawn from the pneumatic chamber, the deformation (strain) of the PDMS membrane with/without the nanofibrous membrane immobilized on the PDMS interstitium was characterized using the same method we reported previously.^48^ Briefly, the deformation of the membranes was captured using a charge-coupled device (CCD) camera (Model DMK 31, The Imaging Source) and the curvilinear profiles of the membranes were obtained using ImageJ (http://rsb.info.nih.gov/ij/index.html).^63^ The theoretical calculation of the linear strain was also detailed previously.^48^

#### Cell growth

The nanofibrous membrane was coated with 50 μg/mL type I collagen (Col I, rat tail, Corning) overnight in the incubator. The cells were seeded at a density of 1 x 10^5^ cells/cm^2^ and cultured in the incubator of 37°C and 5% CO_2_. The cells were cultured under static condition for 1 day, and then subjected to mechanical stretch continuously for at least 5 days and culture medium were refreshed every other day.

#### RNA sequencing of cells cultured in different conditions

For RNAseq experiments, A549 cells were grown on biomimetic substrates or tissue culture plastic. The cells were seeded at 100000 cells/ml density. Following 7 days of culture trypsinized the cells, neutralized with PBS, and extracted RNA using the PURELINK RNA MINI KIT (ThermoFisher, 12183018A) following manufacturer guidelines. For RNA extraction steps, all surfaces were cleaned with RNAse away to minimize degradation.

After purification, the quality of RNA was assessed using both a nanodrop and a tape station 4200 (Agilent) to measure the 260/280 absorbance along with the RNA integrity. Only RNA with RNA integrity (RIN) of more than 9.5 and a concentration of more than 40 ng/μL were assessed for RNAseq. As the samples contained total RNA, mRNA enrichment was done before proceeding to create cDNA libraries. Both steps used the cDNA library TruSeq standard total RNA protocol from Illumina. In this process, TruSeq DNA/RNA indexes from Illumina kit were used as adaptors. After cDNA library preparation, samples were quantified using the TapeStation 4200 with high-sensitivity D1000 tape and D1000 reagents. Samples were normalized with nuclease-free water, to ensure equal concentration of each sample. Diluted samples were pooled, which allowed for maximization of throughput or sequencing, and then were sequenced using NextSeq 500 machine with NextSeq 75bp High-Output kit (Illumina). 400 million reads were performed across the 12 samples.

#### Transcript data analysis

The transcriptomics data was converted into FASTQ format with adaptors trimmed. Analysis was performed using Galaxy (Galaxy Project). Lanes for each sample were combined using Concatenate datasets to create a forward and reverse read file. Sequences were then trimmed with trimmomatic to remove low quality reads. Quality was confirmed using FastQC. HISAT2 function was used to align reads (forwards and reverse) to the Human Genome (hg38). To count the expression of each gene, featureCounts function was used. Based on our experiment the differential expression of genes was the main goal. To observe the differentially expressed genes between our samples the edgeR function was used. The results of this function were the normalized counts of differentially expressed genes for each condition and their comparisons. These results were used for further analysis in RStudio and g-profiler.^64^

Statistically significantly differentially expressed genes (DEGs) were identified (log_2_ fold change <0.5 and false discovery rate (FDR) < 0.05; an adjusted *p*-value). The upregulated and down regulated genes were separated and analyzed for GO in biological processes and KEGG pathways (g-profiler). For GO analysis, the DEGs, both upregulated and downregulated, were uploaded to g-profiler. Since many GO pathways were identified that had close relationships, we only present clustered results that summarize these combined pathways. Only pathways with adjusted *p*-values <0.05 were considered significant. For KEGG and GO analysis, genes were uploaded to g-profiler and significantly enriched pathways (adjusted *p* values <0.05) were identified and clustered results are reported.

#### Immunofluorescent staining of cells cultured in different conditions

We performed immunofluorescence staining on the cells to observe their expression and localization under different mechanical conditions. Cells cultured in dynamic culture interstitium with either soft or stiff matrix and tissue culture plastic were rinsed 3X with 1x Dulbecco’s phosphate buffered saline (DPBS) (Formulation: 200 mg/L KCl and KH_2_PO_4_, 8000 mg/L NaCl, 2160 mg/L Na_2_HPO_4_∗7H_2_O, pH 7–7.6) (BioWhittaker, Lonza) upon reaching confluency. The cells were then fixed with 4% formaldehyde (36.5–38% Formaldehyde solution in H_2_O, Sigma) for 10 min at 37°C in the same culture device. The fixed cells were blocked overnight with 2% Bovine Serum Albumin (Protease free powder) (Fisher Bioreagents) in 1x DPBS (BioWhittaker, Lonza) at 4°C. The blocking solution was removed the next day, and two different primary antibody mixes were added in with 1% Bovine Serum Albumin in DPBS. Specifically, Chondroitin Sulfate Monoclonal Antibody (CS-56) (Invitrogen by Thermo fisher Scientific) (1:500 dilution) and Recombinant Anti-Syndecan-1 antibody [EPR6454] (abcam) (1:500) were used for chondroitin sulfate staining, and Ab-Heparan Sulfate (JM-403) (AMSBIO) (1:500 dilution) and Recombinant Anti-Syndecan-1 antibody [EPR6454] (abcam) (1:500) were used for heparan sulfate staining of cells cultured in both soft and stiff substrates. The cells were incubated overnight at 4°C for primary antibody attachment and rinsed 3X with DPBS the next day before adding secondary antibody and counterstain. Goat anti-Rabbit IgG (H + L) Highly Cross-Adsorbed Secondary Antibody, Alexa Fluor 546 (Invitrogen by Thermo Fisher Scientific) (1:500) was added as a secondary antibody for Recombinant Anti-Syndecan-1 antibody [EPR6454] (abcam). In parallel, Alexa fluor 594 goat anti-mouse IgM μchain (Invitrogen by Thermo Fisher Scientific) secondary antibody (1:1000) was added to bind with either chondroitin sulfate or heparan sulfate primary antibody. All secondary antibodies and counterstain DAPI (1:2000) were prepared in a similar 1% Bovine Serum Albumin solution and the cells were incubated for an hour at room temperature in dark. The stained cells were rinsed with 1x DPBS 3–4 times and left in DPBS overnight. The chips were mounted on coverslips using diamond mount. Zeiss LSM 710 Confocal Scanning Microscope with Airyscan was used to image the cells. These experiments were replicated twice to confirm the glycocalyx localization.

#### Peptide lipid conjugate synthesis

Peptide sequences CERRIWFPYRRF (Chondroitin sulfate C recognition),^57^ CRTPPESYASVR (Chondroitin sulfate A recognition),^54^ CGRKKRRQRRRPQ (Heparan sulfate recognition)^58^ purchased from Genscript with >98% purity, terminated with cysteines were reacted with twice the molar amount of 1, 2-Distearoyl-*sn*-glycero-3-phosphoethanolamine-Poly (Ethylene glycol) (DSPE-PEG20kD) maleimide in anhydrous DMSO with triethylamine overnight at 4°C (Figure S4). Samples were purified on an Accela ultra-high performance liquid chromatographer with a gradient of triethylammonium acetate (TEAA) buffer (0.05 M; pH 7.0) to 95% acetonitrile with TEAA buffer (0.05 M; pH 7.0) on a biobasic C18 column (Thermo Electron Corporation). The concentration of peptide conjugates was determined by the standard Bicinchoninic acid (BCA) assay using Pierce BCA Protein Assay Kit (Thermo Fisher Scientific). To confirm peptide lipid conjugation, peptide only controls were run alongside peptide lipid conjugates in a hand casted 20% polyacrylamide gel (Figure S5A). To prepare resolving gel, 4mL of SureCast 40% (w/v) Acrylamide (29:1 acrylamide: bis-acrylamide) (Invitrogen by Thermo Fisher Scientific), 80ul of 10% solution of SureCast Ammonium Persulfate (APS) (Invitrogen by Thermo Fisher Scientific), 2mL Resolving Buffer (Invitrogen by Thermo Fisher Scientific), 8ul Tetramethylethylenediamine (TEMED) (Fisher Bioreagents) and 1.9mL deionized water was mixed and poured into vertical gel casting setup with glass plates. The stacking gel was added by mixing 300ul of SureCast 40% (w/v) Acrylamide (29:1 acrylamide: bis-acrylamide) (Invitrogen by Thermo Fisher Scientific), 30ul of 10% solution of SureCast Ammonium Persulfate (APS) (Invitrogen by Thermo Fisher Scientific), 750ul Stacking Buffer (Invitrogen by Thermo Fisher Scientific), 3ul Tetramethylethylenediamine (TEMED) (Fisher Bioreagents) and 1.92mL deionized water. The setting time for the gel was 30 min. The electrophoresis ran for 40 min at 200V to ensure complete running of load through the gel. The gel was then stained with Coomassie blue staining solution for 2 h at room temperature. The staining solution was prepared using 0.125% (w/v) Brilliant Blue G-250 (Fisher Bioreagents) in a 50% (v/v) methanol (Fisher Chemicals), 40% (v/v) deionized water and 10% (v/v) glacial acetic acid (Fisher Chemicals) solution. It was destained overnight in the same solution without the Coomassie blue and excess deionized water with 1:2 solution: water ratio to prevent it from drying out. The Gel was imaged in ChemiDoc MP Gel Imaging System (BioRad Laboratories Inc). To further confirm successful synthesis of peptide conjugates, they were reacted with 10:1 Methyltetrazine-sulfo-N-Hydroxysuccinimide ester (Met-NHS) (Click Chemistry Tools): Peptides for an hour at room temperature in Dulbecco’s Phosphate Buffered Saline (DPBS) (BioWhittaker, Lonza). The conjugates were further incubated with 1.1:1 Cyanine 5 *trans*-cyclooctene (AAT Bioquest): Met-NHS for a minute and run through NuPAGE 4–12% Bis-Tris premade polyacrylamide gel (Invitrogen by Thermo Fisher Scientific) (Figure S5B). The electrophoresis ran for 1 h at 120V to ensure complete running of load through the gel. The Gel was imaged in ChemiDoc MP Gel Imaging System (BioRad Labnoratories Inc). The mini gel tank (Invitrogen by Thermo Fisher Scientific) and PowerEase 300W (Life Technologies) system were used for running both of the electrophoresis.

#### Liposome preparation

Liposomes were prepared following the widely used thin film technique with 58 mol % 1,2-dioleoyl-*sn*-glycero-3-phosphocholine (DOPC; Avanti Polar Lipids) and 42 mol % cholesterol (Sigma Aldrich). Liposomes were synthesized by forming lipid cakes by removing chloroform under vacuum for >30 min followed by 1h on a 4.5 L, −105°C lyophilizer (Labconco). Lipids were reconstituted in 1x PBS and sonicated for 20 min. Liposomes were systematically sized by extruding through a 100 nm membrane for 15–20 passes (Nuclepore Track-Etch Membrane, Cytiva). To label liposomes for flow cytometry analysis, 0.1 mol % of 1,1′-dioctadecyl-3,3,3′,3′-tetramethylindodicarbocyanine, 4-chlorobenzenesulfonate salt (DiD; DiIC18(5)) (Invitrogen), a highly lipophilic dye, was mixed with the lipid formulation before lipid cake formation.^65^^,^^66^^,^^67^^,^^68^

#### Peptide decorated liposome preparation and characterization

The number of liposomes in 1mL solution were calculated using the size of liposome and quantity of DOPC and cholesterol. From the concentration of peptide lipid conjugates, we quantified the number of peptide-lipid conjugates and added 300 conjugates per liposome vesicle from our stock solution. Liposomes and peptide conjugate solution were incubated overnight at 4°C in 1 x PBS to allow insertion into the lipid bilayer. Peptide conjugated liposomes were spun down in a 10K MWCO spin filter (Thermo Fisher) using Sorvall Legend Micro 17R centrifuge (Thermo Scientific) at 13,300rpm for 2 min to remove smaller sized peptide lipid micelles. Subsequently, Pierce Quantitative Fluorometric Peptide Assay Kit (Thermo Scientific) was utilized to measure the number of attached peptides following manufacturers protocol. Specifically, a set of standard solutions with different dilutions, sample solutions of modified and bare liposomes in their original dilutions and blank solutions were plated in a 96 well plate, mixed with assay reagents as specified in the protocol and incubated for 30 min at room temperature. Fluorescence intensity was measured using Ex/Em at 390nm/475nm in Cytation 5 imaging reader (BioTek). The number of peptides were calculated from the standard curve generated using the fluorescence intensity and concentration of standard dilutions (Figure S7). Fluorescence intensity of bare liposomes measured were subtracted from the intensity of modified liposomes as baseline correction. Similarly, Phosphatidylcholine Assay Kit (ABCAM) was used to quantify lipid content for normalization following standard fluorometric assay protocol. A 96 well plate was used to lay out the standard dilutions, samples and background control which were mixed with reaction mix prepared from the assay kit specifications. It was then incubated at room temperature for 30 min and fluorescence intensity was measured at Ex/Em of 535nm/587nm, followed by calculations to quantify lipid content in the sample solutions.

Dynamic Light Scattering (DLS) was performed on liposomes with a Zetasizer S (Malvern Instruments). The liposome samples were diluted (1:4) in deionized water (dispersant) and loaded in folded capillary zeta cell (Malvern Panalytical) disposable cuvettes for zeta potential analysis. The cuvettes were then loaded in Zetasizer (Malvern Panalytical) and zeta potential distribution was obtained at room temperature. For morphological analysis, liposome samples were prepared by reconstituting the lipid cake (see liposome preparation step) in HEPES buffer (10mM of HEPES sodium salt (Fisher Bioreagents) with 150mM of NaCl (Fisher Bioreagents) in Deionized water). Formvar carbon 400 mesh copper grid (Electron Microscopy Sciences) was selected for negative staining of liposome. The grid was glow discharged (Pelco easiGlow) and negative staining was done following the inversion parafilm method. Specifically, the glow discharged grid was floated on top of 5 μL of the sample liposome solution drop on a parafilm for 10 s. It was rinsed with 10 μL drop of milli-q water in a similar manner 3 times for 10s each followed by staining with 5 μL 2% uranyl acetate (Electron Microscopy Sciences) drop on the parafilm for 10s. Liposome structures were then imaged with a transmission electron microscope (FEI TecnaiG2 Spirit Biotwin).

#### Liposome uptake by cancerous lung epithelium cells

Lung Epithelium cell line A549 were incubated with 0.15 nM Chondroitin sulfate A, Chondroitin sulfate C and Heparan sulfate targeting peptides decorated liposomes (target of 300 ligands/liposome) along with unmodified controls for 4 h in DMEM. The incubation was initiated after reaching monolayer confluency of cells on their substrates. Uptake efficiency was observed for soft, stiff matrices in both static and dynamic conditions and compared with plastic control. Uptake experiments were performed twice with triplicate samples each time.

#### Flow cytometry of liposome uptake

To quantify the accumulation of the peptide decorated liposomes inside the cells, flow cytometry was performed after 4-h incubation of the cells with liposomes. Cells were collected by trypsinization after 4 h, rinsed 3 times with 1x Dulbecco’s phosphate buffered saline (DPBS) (Formulation: 200 mg/L KCl and KH_2_PO_4_, 8000 mg/L NaCl, 2160 mg/L Na_2_HPO_4_∗7H_2_O, pH 7–7.6) (BioWhittaker, Lonza) fixed with 3.7% paraformaldehyde solution prior to running in flow cytometer (Guava easyCyte 6HT, Luminex). The fixing solution was prepared by mixing 10% formaldehyde (36.5–38% Formaldehyde solution in H_2_O, Sigma) and 89% DPBS. 1% 1.5M NaCl solution prepared with Sodium Chloride crystals (Fisher Bioreagents) in H_2_O was added to balance the pH level of the solution. Forward and side scatter density plots were used as a gating strategy to identify cell population based on their size.

### Quantification and statistical analysis

All statistical analysis was performed with GraphPad PRISM 9 (GraphPad Software, Inc.). Figure 2 contains *n* = 3 biological replicates. Datasets were assessed with one-way ANOVA followed by a Tukey HSD *post hoc* analysis (Figure 5A), a two-way ANOVA followed by a Tukey HSD *post hoc* analysis (Figure 5B), or Welch’s t-test (Figure 5C). Significance thresholds were *α=0.05.* Statistical parameters are as follows: Figure 4 (A: F = 95.87 B: F_interaction_ = 10.3, F_column_ = 44.08, F_row_ = 28.04; C: F = 1.937). Significance levels (*p* values) are indicated in legends of relevant figures, ∗, *p* < 0.05; ∗∗, *p* < 0.01; ∗∗∗, *p* < 0.001; ∗∗∗∗, *p* < 0.0001. All data points are shown along with mean values are shown for all figures in Figure 5; there are also *n* = 9 biological replicates; except control that is *n* = 6 for Figure 5A, *n* = 3 biological replicates and 3 technical replicates for Figure 5B, and *n* = 3 biological replicates for Figure 5C.

## Supplemental information

Document S1. Document S1. Figures S1–S8 and Tables S9 and S10

Table S1. Upregulated GO and KEGG pathways for TC plastic - stiff biomimetics including gene intersections, related to Figure 2 and Tables 1 and 3

Table S2. Downregulated GO and KEGG pathways for TC plastic - stiff Biomimetics including gene intersections, related to Figure 2 and Tables 2 and 3

Table S3. Upregulated GO and KEGG pathways for TC plastic - soft biomimetics including gene intersections, related to Figure 2 and Tables 1 and 3

Table S4. Downregulated GO and KEGG pathways for TC plastic - soft biomimetics including gene intersections, related to Figure 2 and Tables 2 and 3

Table S5. Upregulated GO and KEGG pathways for TC plastic - overlapping stiff/soft biomimetics including gene intersections, related to Figure 2 and Tables 1 and 3

Table S6. Downregulate GO and KEGG pathways for TC plastic - overlapping stiff/soft biomimetics including gene intersections, related to Figure 2 and Tables 2 and 3

Table S7. Upregulated GO and KEGG pathways for stiff biomimetics - soft biomimetics including gene intersections, related to Figure 2 and Tables 1 and 3

Table S8. Upregulated GO and KEGG pathways for stiff biomimetics - soft biomimetics including gene intersections, related to Figure 2 and Tables 2 and 3
