# Supplementary material for: Targeting nanoparticles to lung cancer-derived A549 cells based on changes on interstitial stiffness in biomimetic models
Source: iScience. 2024 Sep 23;27(10):111015. doi: 10.1016/j.isci.2024.111015 (PMC11492096; doi:10.1016/j.isci.2024.111015)
Supplement: Document S1. Document S1. Figures S1–S8 and Tables S9 and S10 [file mmc1.pdf]

iScience, Volume *27*

Supplemental information

Targeting nanoparticles to lung cancer-derived A549 cells based on changes on interstitial stiffness in biomimetic models

Afia Ibnat Kohon, Kun Man, Ala Hessami, Katelyn Mathis, Jade Webb, Joanna Fang, Parsa Radfar, Yong Yang, and Brian Meckes

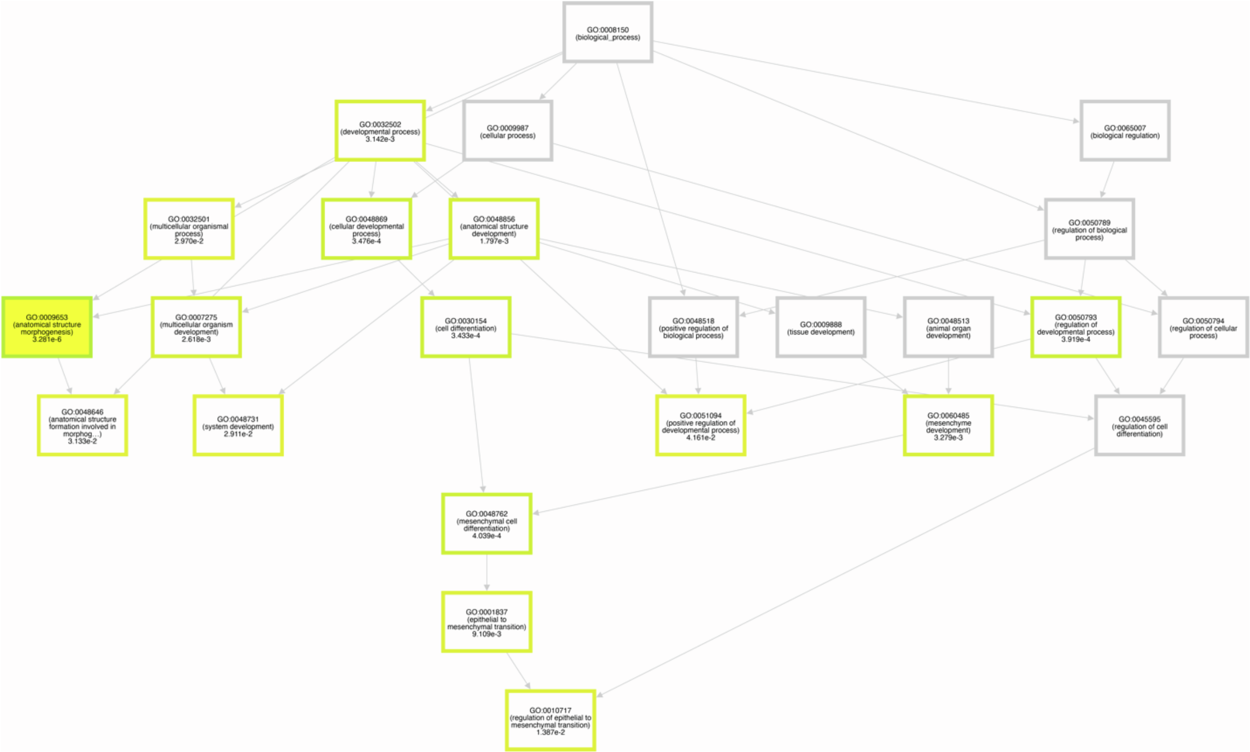

Figure S1: CNET plot showing related terms contributing to the GO biological process of anatomical structural morphogenesis that is downregulated compared to TC plastic compared to soft biomimetic substrates. Related to Table 2

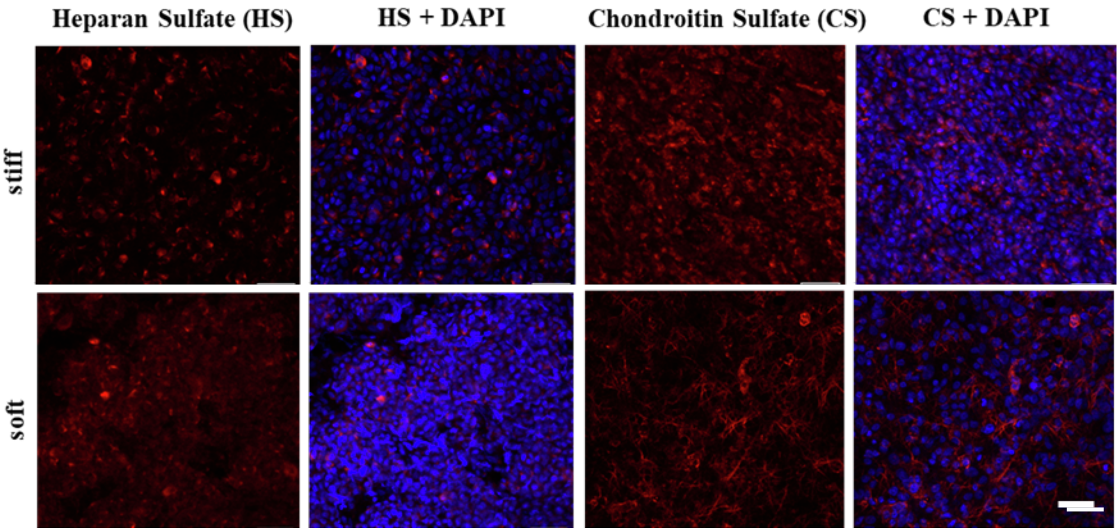

Figure S2: Effect of substrate mechanics on A549 glycocalyx proteoglycan and glycosaminoglycan localization when grown on biomimetics. Immunofluorescent staining of epithelial cells for chondroitin sulfate and heparan sulfate (red) with DAPI (blue) as a counterstain, cultured in soft and stiff matrix with 3-Dimensional stretch. Image captured in Z-stack 10X magnification and projected in maximum intensity. Scale bar 50μm. Related to Figure 3.

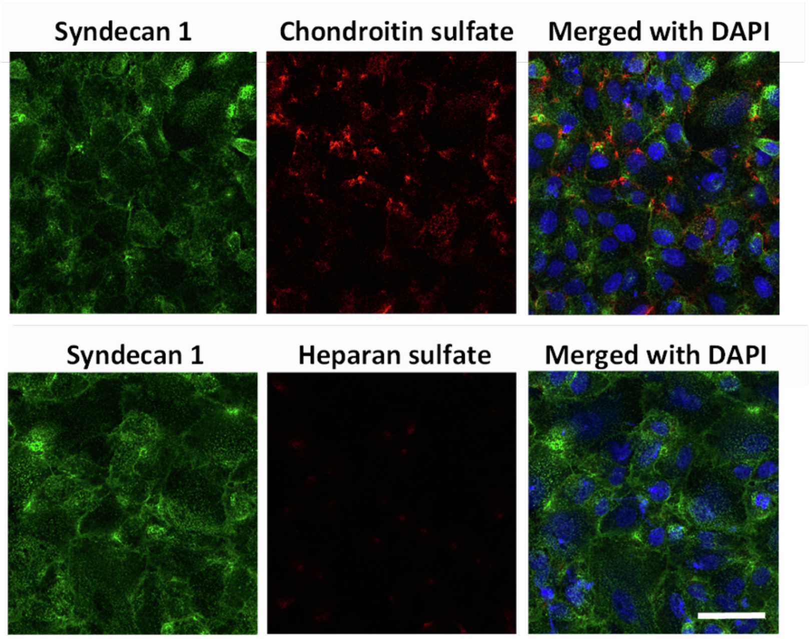

Figure S3: Glycocalyx proteoglycan and glycosaminoglycan production and localization in conventional tissue culture plastic condition. Immunofluorescent staining of epithelial cells for syndecan 1 (green), chondroitin sulfate and heparan sulfate (red) with DAPI (blue) as a counterstain, cultured in soft and stiff matrix with 3-Dimensional stretch. Image captured in Z-stack 40X magnification and projected in maximum intensity. Scale bar 100µm. Related to Figure 3.

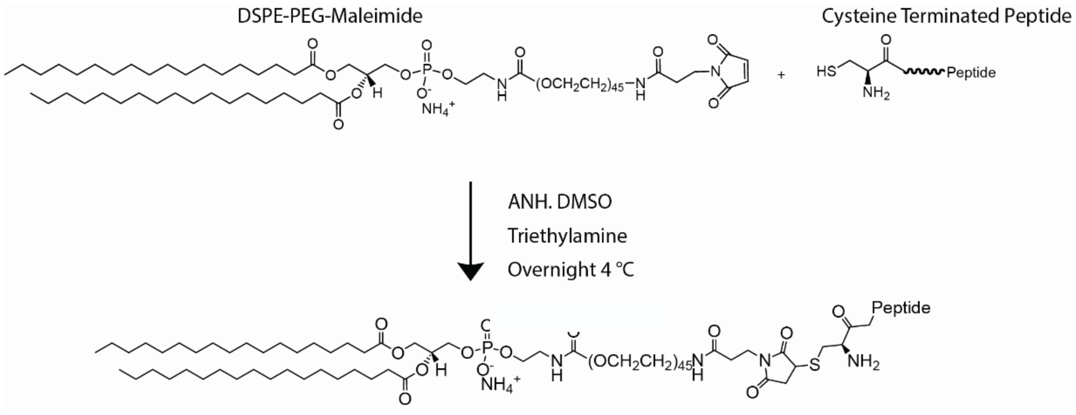

Figure S4. Schematic depicting peptide conjugation to lipids. Cysteine terminated peptides are reacted with DSPE-PEG-Maleimide in anhydrous DMSO overnight. Related to Star Methods.

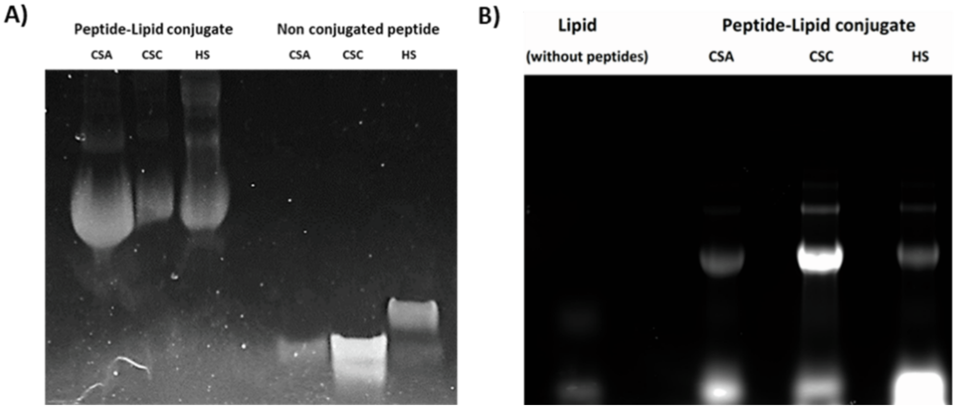

Figure S5. Electrophoresis of glycocalyx targeting peptide-lipid conjugates confirms synthesis of conjugates. (A) Polyacrylamide gel electrophoresis followed by coomassie blue staining of CSA, CSC, and HS targeting peptide-lipid conjugates and non-conjugated peptides, confirmed the conjugation through the observed size shift of the peptide-lipid conjugate bands in comparison to the unconjugated peptides. **(B)** Polyacrylamide gel electrophoresis of CSA, CSC and HS targeting peptide-lipid conjugates after reacting with N-Hydroxy succinimide ester (NHS) methyl tetrazine and trans-cyclooctyne fluorophore confirmed the presence of peptide lipid conjugate bands. No conjugation band was observed after a similar reaction with lipids. Related to Figure 4 and Star Methods.

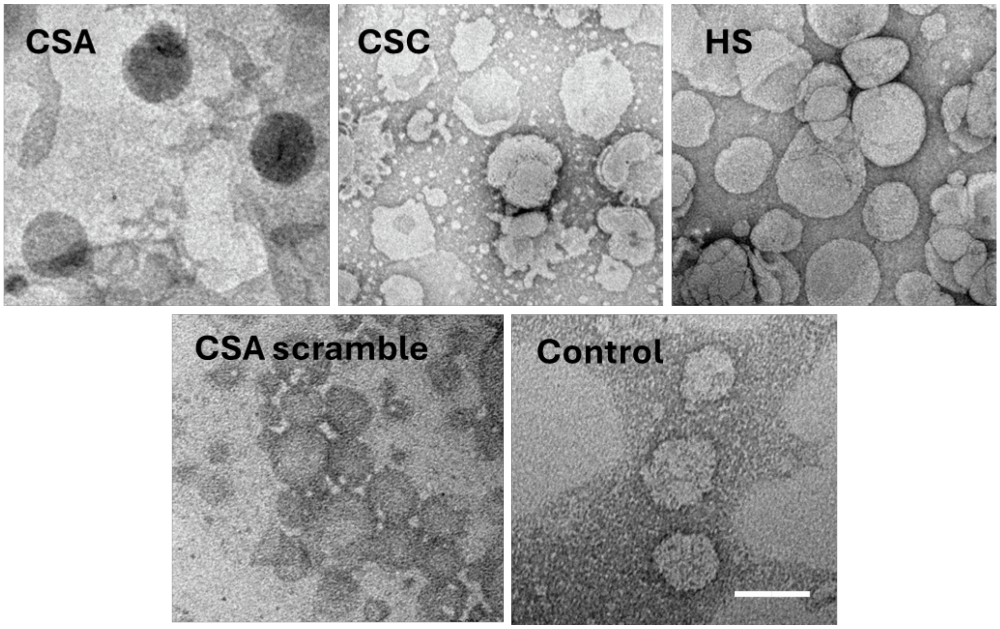

Figure S6. Transmission Electron Microscopy (TEM) images showing liposome morphology. Negative staining of liposomes modified with chondroitin sulfate A (CSA), chondroitin sulfate C (CSC), heparan sulfate (HS), chondroitin sulfate A scramble (CSA scramble and bare liposomes (Control) observed at low magnification. Scale bar 100 nm. Related to Figure 4.

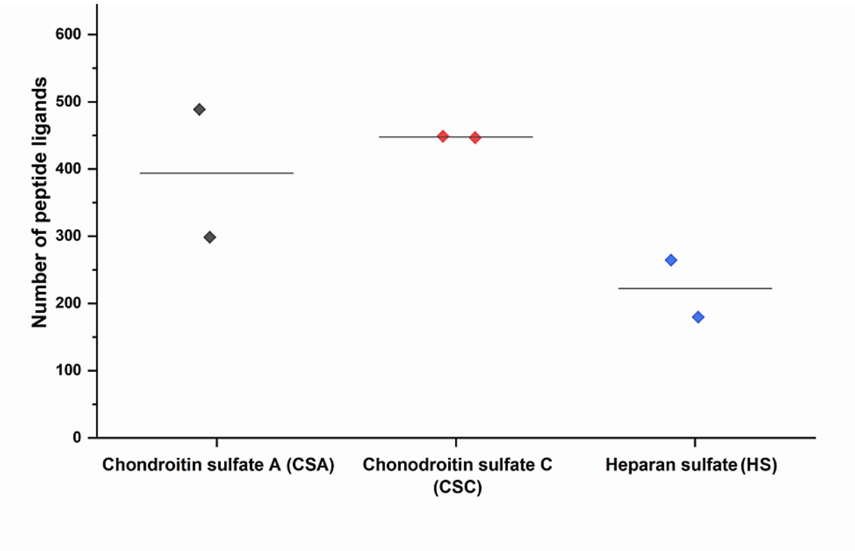

Figure S7. Number of peptides attached on liposome surface. Fluorometric peptide assay confirmed the presence and number of peptides on each liposome surface for chondroitin sulfate A, chondroitin sulfate C and heparan sulfate targeting peptides. Related to Figure 4 and Star Methods

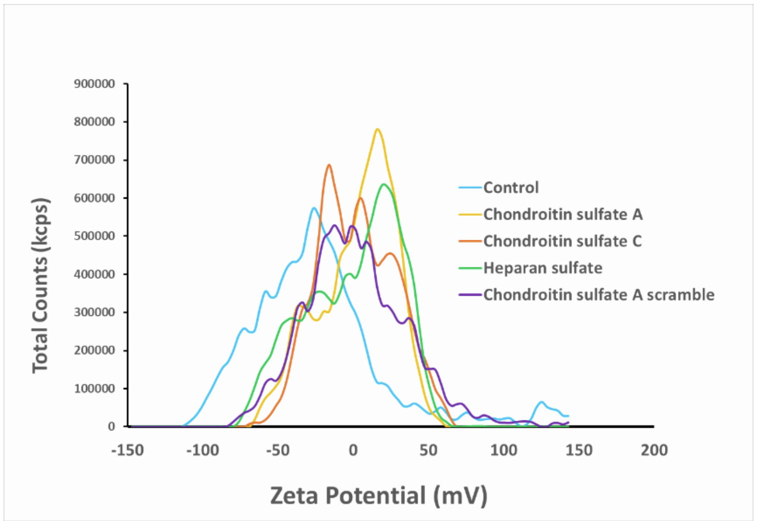

Figure S8. Zeta potential distribution of liposomes. An overall neutral distribution of charges of liposomes modified with targeting peptides chondroitin sulfate A (CSA), chondroitin sulfate C (CSC), heparan sulfate (HS) and chondroitin sulfate A scramble (CSA Scramble) and bare liposomes (control). A small positive shift was observed from bare liposomes when positively charged peptides were attached on the surface. Related to Figure 4.

Table S9. Size and polydispersity index of peptide micelles and peptide targeting liposomes. Related to Figure 4

Sample	Size (nm)	Polydispersity index
CSA micelle	24.67	0.427
CSC micelle	30.13	0.253
HS micelle	24.44	0.245
CSA liposome	111.6	0.099
CSC liposome	106.7	0.100
HS liposome	104.7	0.102
Bare liposome	84.07	0.122

Table S10. Zeta potential average of bare and peptide decorated liposomes. Related to Figure 4 and Star Methods

Sample	Zeta Potential (mV) (mean)
Bare liposomes	3.646
CSA liposome	0.6416
CSC liposome	0.914
HS liposome	-0.04444
CSA scramble liposome	1.412
